# Valorization of Onion-Processing Waste: Digestive Fate, Bioavailability, and Cellular Antioxidant Properties of Red and Yellow Peels Polyphenols

**DOI:** 10.3390/antiox15010007

**Published:** 2025-12-20

**Authors:** Anna Rita Bavaro, Isabella D’Antuono, Angelica Bruno, Francesca Anna Ramires, Vito Linsalata, Gianluca Bleve, Angela Cardinali, Antonella Garbetta

**Affiliations:** 1 National Research Council, Institute of Sciences of Food Production (CNR-ISPA), 70126 Bari, Italy; isabella.dantuono@cnr.it (I.D.); angelicabruno@cnr.it (A.B.); vito.linsalata@cnr.it (V.L.); angela.cardinali@cnr.it (A.C.); 2 National Research Council, Institute of Sciences of Food Production (CNR-ISPA), 73100 Lecce, Italy; francesca.ramires@ispa.cnr.it (F.A.R.); gianluca.bleve@cnr.it (G.B.)

**Keywords:** onion peel, *Allium cepa*, polyphenols, antioxidant activity, bioavailability, Caco-2 cells, in vitro digestion, flavonoids

## Abstract

Onion (*Allium cepa* L.) peels represent a major agro-industrial by-product and are a rich source of polyphenols, with recognized antioxidant properties. This study compared the polyphenolic profile of two onion cultivars peels: red “*Rossa di Tropea*” and yellow “*Recas*”. Their digestive stability, intestinal bioavailability, and antioxidant activity were evaluated. Hydroalcoholic extracts were characterized by HPLC-DAD, subjected to a static gastrointestinal digestion model, and assessed for transport across differentiated Caco-2 monolayers. Antioxidant properties were determined using DPPH, FRAP, Cellular Antioxidant Activity (CAA), and intracellular glutathione (GSH) assays. Red peels contained a higher total polyphenol content (28.44 mg/g DW) than yellow peels (15.61 mg/g DW), including anthocyanins uniquely present in the red cultivar. Digestive stability varied markedly between cultivars, with yellow peels showing greater intestinal recovery (72.7%) than red peels (49.1%). Glycosylated flavonols were more stable and exhibited moderate intestinal transport (Papp = 1.1–9.9 × 10^−6^ cm·s^−1^), whereas quercetin aglycone showed low permeability. Red peel extracts demonstrated stronger chemical antioxidant activity, while yellow peels were more effective in cell-based assays, displaying higher CAA values and inducing a pronounced increase in intracellular GSH. Overall, onion peel extracts exhibit promising antioxidant and biological properties. However, their limited bioavailability highlights the need for formulation strategies to enhance gastrointestinal stability and intestinal uptake, supporting their potential use as sustainable functional ingredients.

## 1. Introduction

Onion (*Allium cepa* L.) is among the most extensively cultivated and consumed vegetables worldwide, valued not only for its culinary versatility but also for its nutritional and medicinal significance. Belonging to the *Amaryllidaceae* family, the onion is a staple ingredient in numerous dietary traditions and is recognized for its rich phytochemical composition, which includes sulfur-containing compounds, flavonoids, and various phenolic metabolites associated with diverse biological activities [1].

Despite its global importance, onion processing generates substantial amounts of waste, particularly the dry outer peels and skins that are usually discarded. In Europe alone, approximately 0.6 million tons of onion peel waste are produced annually [2]. Although unsuitable for animal feed due to its intense aroma and low digestibility, and not ideal as fertilizer, onion peels represent a valuable source of bioactive compounds, especially polyphenols. Their valorization aligns with the rising interest in sustainable food production and circular bioeconomy approaches.

The phytochemical composition of onion peels is influenced by multiple factors, including cultivar, geographical origin, agronomic practices, and environmental conditions [3]. Peel color is strongly associated with phenolic composition: yellow onions are typically rich in flavonoids, particularly quercetin and its glycosides, whereas red onions contain significant levels of anthocyanins responsible for their pigmentation [4,5]. These compounds accumulate predominantly in the outer epidermal layers of the bulb and serve protective functions against abiotic and biotic stressors, such as ultraviolet (UV) radiation and microbial pathogens [6,7].

Quercetin is one of the most abundant flavonols in onions and occurs both in free derivatives and as conjugated derivatives, such as quercetin-3-O-glucoside, quercetin-4′-O-glucoside, and quercetin-3,4′-O-diglucoside. Additional phenolic constituents, including protocatechuic acid, *p*-coumaric acid, kaempferol, and isorhamnetin, have also been identified in onion peels [8,9]. These phytochemicals exhibit a broad spectrum of bioactivities, including antioxidant, anti-inflammatory, anticancer, and neuroprotective effects, underscoring their potential for nutraceutical, pharmaceutical, and functional food applications.

Increasing attention has been devoted to the incorporation of onion peel extracts into food matrices to enhance their functional properties. Previous studies have shown that the addition of onion polyphenols can elevate antioxidant capacity in bakery products and fermented foods, while also exhibiting anti-obesity and anticancer properties in experimental systems [10]. As consumer awareness of the relationship between diet and health grows, the use of flavonoid-rich extracts to formulate value-added functional foods continues to expand.

The biological efficacy of onion polyphenols is closely linked to their antioxidant potential. These compounds directly neutralize reactive oxygen species (ROS) and chelate metal ions that catalyze oxidative reactions, thereby preserving cellular integrity. Furthermore, polyphenols can modulate endogenous antioxidant defense systems by influencing the expression or activity of key enzymes, such as superoxide dismutase catalase and glutathione-related enzymes [11,12,13]. Through these mechanisms, they play a significant role in maintaining redox homeostasis and mitigating inflammatory processes via modulation of pathways associated with cyclooxygenase and nitric oxide synthase [14].

However, beyond their intrinsic chemical reactivity, the functionality of polyphenols is largely determined by their bioaccessibility and bioavailability. These parameters are influenced by structural features such glycosylation, acylation, and polymerization, which affect their digestive stability and intestinal absorption [15]. Although dietary polyphenols demonstrate good bioaccessibility, their systemic bioavailability is often limited due to restricted intestinal uptake [16]. Nevertheless, unabsorbed polyphenols are metabolized in the colon by the gut microbiota into low-molecular-weight derivatives with improved absorption and potentially enhanced biological activity [17,18].

Given these considerations, onion peels represent a promising yet underutilized source of health-promoting compounds. Their high polyphenol content and demonstrable biological activities make them strong candidates for use in nutraceutical formulations and functional food enrichment.

The main objective of the present study was to characterize the polyphenolic profile of two *Allium cepa* L. cultivars—red “*Rossa di Tropea*” (PGI, Italy) and yellow “*Recas*” (Spain)—representing commercially relevant varieties in the Mediterranean region. Specifically, the study aimed to (i) evaluate the digestive stability and bioaccessibility of their polyphenols using an in vitro gastrointestinal digestion model; (ii) assess intestinal absorption using the Caco-2 cell monolayer model as a predictor of bioavailability, including calculation of the apical-to-basolateral permeability coefficient; and (iii) determine the antioxidant potential of hydroalcoholic extracts from both cultivars by examining their radical-scavenging activity and their capacity to modulate endogenous antioxidant defenses in intestinal epithelial cells.

## 2. Materials and Methods

### 2.1. Raw Feedstock

Red onion peels of PGI “*Rossa di Tropea*” were supplied by the Italian company Azienda Agricola Veltri srl (Catanzaro, Italy), and yellow onion peels of “*Recas*” were provided by PROCECAM (Association of Onion Producers of Castilla-La Mancha, Villarrobledo, Spain). Both peel types were processed into powder following the procedure described by Ramires et al. [5].

### 2.2. Polyphenol Extraction and Characterization

Polyphenols were extracted from red and yellow onion peels using a 60% ethanol solution (ethanol/water, 60:40 (*v*/*v*)) acidified with 0.5% (*v*/*v*) trifluoroacetic acid (TFA). The extraction followed the method described in Ramires et al. [5], maintaining a solid-to-solvent ratio of 1:60. Specifically, approximately 0.50 g of each dried sample was mixed with 20 mL of solvent. The mixtures were constantly stirred (150 rpm) for 30 min at room temperature. The extracts were collected after centrifugation at 4500× *g* for 10 min. The extraction process was performed twice by adding 10 mL of fresh solvent to the pellets, and the resulting supernatants were combined for subsequent analysis. Aliquots of the hydroalcoholic extracts were filtered through 0.45 μm filters and analyzed using a High-Performance Liquid Chromatography with Diode Array Detection (HPLC-DAD) system (Agilent 1260 Infinity Series, Agilent Technologies, Palo Alto, CA, USA), operated with Agilent OpenLab CDS ChemStation Software version C.01.05. The system was equipped with a 1260 HIP Degasser, G1312B Binary Pump, G1316A Thermostat, and G4212B DAD Detector. Separation was achieved using a Phenomenex Luna C18 column (5 μm, 4.6 × 250 mm; Phenomenex, Torrance, CA, USA). The mobile phases were methanol (solvent A) and acetic acid/water (5:95 *v*/*v*) (solvent B), and the gradient elution profile was as follows: 0–25 min, 15–40% A; 25–30 min, 40% A (isocratic); 30–45 min, 40–63% A; 45–47 min, 63% A (isocratic); 47–52 min, 63–100% A; and 52–56 min, 100% A (isocratic). The flow rate was maintained at 1 mL/min. Phenolic compounds were recorded at three wavelengths, in particular, at 280 nm (phenolic acids), 360 nm (flavonoids), and 530 nm (anthocyanins), and the main polyphenols were identified by comparing the spectra and retention time of the pure available standards. In the absence of reference standards, taxifolin glycoside was quantified as taxifolin equivalent, quercetin glycoside as quercetin 3-glucoside equivalent, and cyanidin glycoside as cyanidin 3-glucoside equivalent. Results were expressed as milligrams of compound per gram of onion peel (mg/g DW).

### 2.3. Evaluation of Onion Polyphenols’ Digestive Stability and Bioavailability

#### 2.3.1. In Vitro Digestion

The digestive stability of polyphenols was assessed using a two-step in vitro gastrointestinal digestion model, as described by Cardinali et al. [19]. Aliquots (3 mL) of aqueous onion peel extracts (500 μg/mL total polyphenols, determined by HPLC-DAD) were diluted with 4.5 mL of 0.9% NaCl at pH 7. The gastric phase was initiated with addition of 0.9 mL of porcine pepsin solution (40 mg/mL) in 0.1 N HCl and adjustment of pH to 2.5 ± 0.1 with 1 N HCl. Samples were incubated at 37 °C for 1 h. Following gastric digestion, the small intestinal phase was initiated by adjusting the pH of gastric digesta to 5.3 with 100 mM NaHCO_3_ and 1 N NaOH, followed by the addition of 2.7 mL of small intestinal enzyme solution (lipase 2 mg/mL, pancreatin 4 mg/mL, and bile 24 mg/mL in 100 mM NaHCO_3_). The final sample pH was adjusted to 6.5 ± 0.1 with 1 N NaOH, the volume was standardized to 15 mL with saline solution (0.9% NaCl), and the sample was incubated at 37 °C for 2 h. After both digestion steps (gastric and small intestinal), the samples were collected and centrifuged (10,000× *g*, 1 h, 4 °C), filtered (0.45 μm), and then analyzed by HPLC-DAD. Digestive stability (%) was calculated by comparing pre- and post-digestion polyphenol content.

#### 2.3.2. Cell Line

The human colon carcinoma cell line Caco-2 (ECACC, Sigma Aldrich, St. Louis, MO, USA) was cultured in 25 cm^2^ flasks at a starting density of 250,000 cells/mL in Dulbecco’s Modified Eagle’s Medium, with 4.5 g/L glucose supplemented with 10% FBS, 1% L-Glutamine, 1% Antibiotic and Antimycotic solution, and 1% non-essential amino acid solution. Cell viability and density were determined using Scepter Cell Counter 2.0. The cells used for experimental protocols showed a mean viability of 90%.

#### 2.3.3. Bioavailability and Uptake of Polyphenols by Caco-2 Cells

To determine how effectively specific polyphenols might be absorbed through the human intestine, an in vitro assay was conducted utilizing the Caco-2 cell line, which, when fully differentiated, mimics the human intestinal barrier, as described by D’Antuono et al. [16]. Caco-2 cells were seeded at a density of 1.2 × 10^5^ cells/mL onto polyethylene terephthalate (PET) track-etched membrane inserts (pore size, 0.4 μm; growth area, 4.2 cm^2^; Falcon, BD), which had been pre-treated with poly-L-lysine (50 μg/mL). The cells were maintained for 21 days in a standard culture condition to ensure complete differentiation into a tight monolayer. The medium in both the apical and basolateral compartments was refreshed twice weekly.

The development of a robust epithelial barrier was confirmed by measuring the Transepithelial Electrical Resistance (TEER) using a volt–ohm meter. Only those cell monolayers demonstrating a barrier integrity value exceeding 700 Ω cm^−2^ were deemed suitable for the transport study. Prior to the experiment, the monolayers were rinsed with a pH 5.5 PBS. The apical chamber then received 2 mL of phenol red-free DMEM containing hydroalcoholic extracts from red and yellow onion peels, adjusted to a final total polyphenol concentration of 60 μg/mL and 30 μg/mL, respectively. The system was incubated at 37 °C, and samples from both the apical and basolateral chambers were collected for 30, 60, 90, and 120 min to track the time-dependent flux of the compounds. These samples were flash-frozen at −80 °C pending High-Performance Liquid Chromatography (HPLC) analysis.

For the assessment of polyphenol accumulation within the Caco-2 cells, the monolayers were first subjected to a sequential wash with PBS pH 5.5, and then with a solution of 0.1% fatty acid-free bovine albumin in PBS pH 5.5. The cells were then mechanically harvested into 1 mL of cold PBS pH 5.5 and stored at −80 °C under a nitrogen atmosphere. Protein quantification of the harvested cells was performed using the standard Bradford method [20]. Polyphenols were chemically isolated from the sonicated Caco-2 cells and the basolateral solutions via a liquid–liquid extraction technique, employing ethyl acetate EtOAc stabilized with 0.01% butylated hydroxytoluene (BHT). This extraction was repeated three times, and the pooled organic layers were dried under vacuum before being dissolved in 200 μL mobile phase for HPLC analysis.

The HPLC results were essential for calculating the Apparent Permeability Coefficient (P_app_), which quantifies the rate of compound transport from the apical (A, donor) side to the basolateral (B, receiver) side in cm s^−1^. The governing equation for this calculation is as follows:

(1) Papp = (dC/dt) V/(C_0_A)

where

dC/dt (polyphenol concentration variations at different times, μg mL^−1^ s^−1^) is the appearance rate of polyphenols in the receiver compartment at 30, 60, 90, and 120 min;
V is the volume of the receiver compartment (3 cm^3^);
C_0_ (μg mL^−1^) is the initial concentration in the donor compartment;
A is the exposed area of the tissue (4.2 cm^2^).

### 2.4. Antioxidant Activity of Onion Polyphenols

#### 2.4.1. DPPH Radical-Scavenging Activity

The antioxidant activity of polyphenols extracts from red and yellow onion peels was quantified using the DPPH (2, 2-diphenyl-1-picrylhydrazyl) radical-scavenging microplate method reported by Bobo-García et al. [21], with some modifications. The polyphenols concentration range for both cultivars was from 6 to 60 µg/mL. Briefly, 20 μL of the diluted sample was mixed with 180 μL of 0.15 mM DPPH solution (methanol/water, 80:20, *v*/*v*) in a 96-well microplate. After 40 min in the dark at room temperature, the absorbance was measured at 515 nm into a Varioskan Flash Spectral Scanning Multimode Reader (Thermo Fisher Scientific, Waltham, MA, USA). Trolox was used as a standard at 10–200 μg/mL to generate a calibration curve. The % DPPH quenched (% scavenging) was calculated as follows:

(2) % DPPH quenched = [1 − (A_sample_ − A_blank_)/(A_control_ − A_blank_)] × 100

where

A_sample_ is the absorbance at 515 nm of 20 μL of extract or standard with 180 μL DPPH solution after 40 min;
A_blank_ is the absorbance at 515 nm of 20 μL of acidified EtOH 60% with 180 μL MeOH 80% after 40 min;
A_control_ is the absorbance at 515 nm of 20 μL of acidified EtOH 60% with 180 μL DPPH solution after 40 min.

Five serial concentration extracts dissolved in acidified EtOH 60% were prepared to obtain the IC_50_ value, defined as the concentration of the test material required to cause a 50% DPPH inhibition. Results were also reported as µmol TE (Trolox equivalent)/g DW (dry weight) according to the formula of Xiao et al. [22].

#### 2.4.2. Ferric-Reducing Antioxidant Power (FRAP)

The ferric ion-reducing antioxidant power (FRAP) assay was performed on polyphenols extracts from red and yellow onion peels according to Firuzi et al. [23], with some modifications. For the assay, we used a freshly prepared reagent containing acetate buffer (300 mM, pH 3.6), 10 mM TPTZ (2,4,6-Tris(2-pyridyl)-s-triazine), and 20 mM FeCl_3_·6H_2_O (10:1:1, *v*/*v*/*v*). In 96-well plates, 25 µL of sample or Trolox standard was mixed with 175 µL of reagent and incubated at 37 °C for 60 min. Absorbance was measured at 595 nm. Solutions of known Trolox concentrations in the range of 1–100 μg/mL were used for calibration. Results were expressed as µmol TE/g DW. The polyphenol concentration quantified by HPLC-DAD, as reported in Section 2.2, ranged from 5 to 25 µg/mL.

#### 2.4.3. MTT Assay

The toxic effect of hydroalcoholic extract of polyphenols from red and yellow onion peels was assessed on proliferation of Caco-2 cells by using a colorimetric MTT assay as previously reported by Minervini et al. [24]. The MTT assay is used to measure cellular metabolic activity as an indicator of cell viability, proliferation, and cytotoxicity. Briefly, Caco-2 cells were seeded at a cell density of 250,000 cells/mL in 96-well plates and exposed for 3 h to polyphenols extracts at concentrations ranging from 8 to 120 µg/mL for red onion and 4 to 60 µg/mL for yellow ones. After incubation, cells were loaded with MTT (5 mg/mL) and incubated for 4 h at 37 °C. Absorbance was measured at 580 nm in a Varioskan Flash Spectral Scanning Multimode Reader (Thermo Fisher Scientific).

#### 2.4.4. Cellular Antioxidant Activity (CAA)

Antioxidant activity of onion polyphenols was evaluated as the ability to reduce induced intracellular ROS using a fluorescent DCFH-DA (2′,7′-Dichlorofluorescein diacetate) probe with CAA assay according to Wolfe and Liu [25], with some modifications, as reported in our previous study [13].

Intestinal cells were stained with this probe, treated with polyphenol extracts, and then exposed to an oxidative stress inductor. Caco-2 cells were seeded at 5 × 10^4^ cells/well on a 96-well white flat-bottom plate and incubated at 37 °C for 24 h. Cells were stained with 5 μM DCFH-DA and incubated for 30 min. Then, cells were treated for 30 min with different extracts at polyphenol concentrations ranging from 0.71 to 28.40 µg/mL for red onion and from 0.50 to 15.05 µg/mL for yellow onions. Finally, cells were treated with cumene hydroperoxide (12.5 μM) as stress inductor for the last 10 min, and the fluorescence was measured every 5 min for 1 h at 37 °C into a Varioskan Flash Spectral Scanning Multimode Reader (Thermo Fisher Scientific) at emission wavelength of 530 nm and excitation wavelength of 485 nm. The quantification of CAA units (the percentage of inhibited ROS production) and the median effective dose (EC_50_) were performed following the mathematical protocols proposed by Wolfe and Liu [25]. CAA values and median effective dose are expressed as mean ± standard deviation of three independent experiments.

#### 2.4.5. GSH Determination

The effect of natural antioxidants on the redox status of intestinal cell lines to evaluate if polyphenols could stimulate antioxidant enzymatic defense of cells will be studied in depth by determining the intracellular reduced glutathione (GSH). Monochlorobimane (MCB) is the most specific probe for GSH determination because its conjugation with GSH forms the highly fluorescent bimane-glutathione adduct by endogenous S-transferase (GST). The levels of intracellular GSH will be measured after cells’ exposure to bioactive compounds. The formed fluorescent MCB-GSH complex will be measured by the fluorimetric technique.

Briefly, intestinal cells were seeded in a 96-well white flat-bottom plate and, after overnight incubation, were supplemented with extracts of red and yellow onion at total a polyphenol concentration ranging from 0.81 to 28.40 µg/mL and from 0.50 to 15.05 µg/mL, respectively, for 1 h [13]. The cells were loaded with MCB (40 μM/well) for 15 min at room temperature in the dark. Thereafter, the formed fluorescent MCB-GSH complex was measured at an excitation wavelength of 395 nm and an emission wavelength of 460 nm with Varioskan Flash Spectral Scanning Multimode Reader (Thermo Fisher Scientific).

#### 2.4.6. Statistical Analysis

Results are reported as mean values ± standard deviation of the mean determined in triplicate from three independent experiments. For digestive stability and antioxidant assays, data were analyzed using an independent-samples *t*-test (*p* < 0.05). For bioavailability and uptake results, data were evaluated by repeated-measures ANOVA, followed by Tukey’s post hoc test (*p* < 0.05). Statistical analyses were performed using Statistica 12.0 (StatSoft Inc., Tulsa, OK, USA) and SigmaPlot 12.0 (Systat Software Inc., San Jose, CA, USA).

## 3. Results and Discussion

### 3.1. Polyphenol Characterization of Red and Yellow Onion Peels

**Figure 1:**
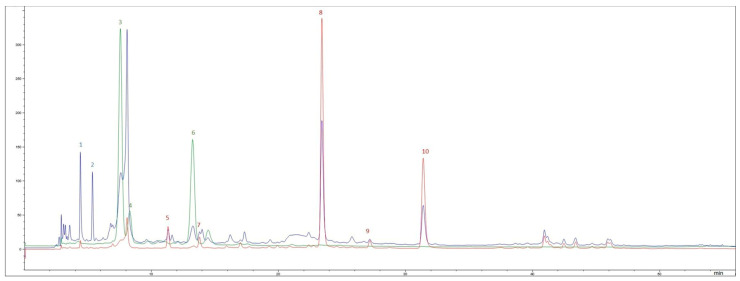
HPLC-DAD chromatograms of the hydroalcoholic extract obtained from red onion peels recorded at 280 nm (red line), 360 nm (blue line), and 530 nm (green line). (1) Taxifolin glycoside, (2) protocatechuic acid, (3) cyanidin 3-glucoside, (4) cyanidin 3-(6″-malonylglucoside), (5) quercetin 7,4′-diclucoside, (6) cyanidin glycoside, (7) quercetin 3,4′-diglucoside, (8) spiraeoside, (9) quercetin glycoside, and (10) quercetin.

**Figure 2:**
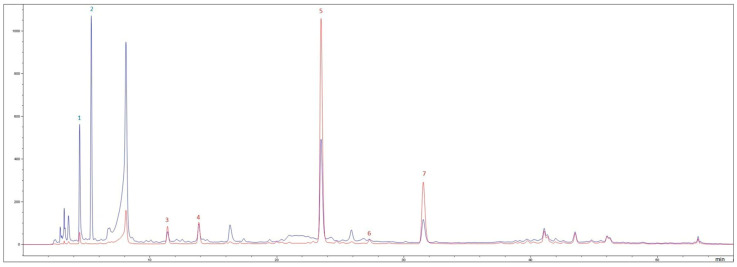
HPLC-DAD chromatograms of the hydroalcoholic extract obtained from yellow onion peels recorded at 280 nm (red line) and 360 nm (blue line). (1) Taxifolin glycoside, (2) protocatechuic acid, (3) quercetin 7,4′-diclucoside, (4) quercetin 3,4′-diglucoside, (5) spiraeoside, (6) quercetin glycoside, and (7) quercetin.

The polyphenolic characterization of red and yellow onion peels, determined by HPLC-DAD (Figure 1 and Figure 2), revealed marked differences in both composition and digestive stability (Table 1 and Table 2).

Red onion peels (Table 1) exhibited a significantly higher content of total polyphenols identified by HPLC-DAD (28.44 ± 1.42 mg/g DW) compared to yellow onion peels (15.61 ± 0.78 mg/g DW; Table 2), confirming their role as a richer source of flavonoids and anthocyanins [5].

It is interesting to underline the presence in the red onion peels of anthocyanins, including cyanidin 3-glucoside, cyanidin 3-(6″-malonylglucoside), and cyanidin glycoside. In red onion peels, quercetin (14.07 ± 0.70 mg/g DW) and spiraeoside (8.08 ± 0.40 mg/g DW) were the predominant compounds, in agreement with the results reported by Celano et al. [26]. Celano et al. [26] and Metrani et al. [27] characterized and compared the flavonoid profiles and biological activities of onion skin wastes from traditional red onion varieties, including the “*Rossa di Tropea*”. They reported that flavonols were the major bioactive constituents among the cultivars studied, with quercetin-4′-glucoside (spiraeoside) and quercetin representing more than 50% of the total identified compounds. It is also worth noting the presence of taxifolin glycoside, a flavanonol with a distinctive structure that has been studied for its broad spectrum of pharmacological activities, including antitumor effects [28]. The amount and relative abundance of onion phenolic compounds have been found to depend strongly on onion variety, harvest year, and meteorological conditions [29].

Yellow onion peels (Table 2) contained high amounts of spiraeoside (8.35 ± 0.42 mg/g DW), quercetin (2.85 ± 0.14 mg/g DW), and protocatechuic acid (2.13 ± 0.11 mg/g DW). Likewise, Paesa et al. [28] characterized the phenolic composition of onion waste extracts from a typical Spanish yellow onion cultivar, reporting a high content of protocatechuic acid and demonstrating the antioxidant and antiproliferative activities of the extract.

The presence of protocatechuic acid in onion peels was also reported by Cattivelli et al. [30], who explained that protocatechuic acid is a well-known quercetin degradation product, resulting from oxidative decarboxylation via nucleophilic attack by oxygen, followed by C-ring cleavage. They hypothesized that a similar mechanism may occur within the food matrix. Our findings confirm previous reports indicating quercetins as the main polyphenols in the outer layers of onions, particularly in red cultivars, due to their higher pigment content and enhanced flavonoid biosynthetic activity [31]. Similarly, Kwak et al. [7] compared the flavonoid content in three different onion varieties (red, yellow, and chartreuse), demonstrating variation in quercetin glycoside derivatives and the associated antioxidant activity.

### 3.2. Digestive Stability of Onion Polyphenols

The digestive stability of individual polyphenols in red and yellow onion peel extracts following simulated two-step gastrointestinal digestion is reported in Table 1 and Table 2, respectively. Overall, total polyphenol recovery decreased during digestion, with greater losses occurring during the intestinal phase in the red onion extract. Specifically, total polyphenol recovery in red onion peels dropped from 63.39% after the gastric phase to 49.09% after intestinal digestion, whereas yellow onion peels showed higher recovery, maintaining 70.92% in the gastric phase and 72.73% after the intestinal phase. This indicates better overall digestive stability of the yellow onion matrix compared to the red one. In both onion varieties, quercetin glycosides exhibited good gastric stability, ranging from 78.8 to 99.5% in red onions and from 69.4 to 97.4% in yellow onions, but their levels decreased significantly during the intestinal phase (30.9–45.6% and 36.8–52.5%, respectively), likely due to pH-induced degradation and enzymatic hydrolysis. In contrast, quercetin aglycone showed the lowest recovery, particularly in yellow onions (3.44% gastric; 15.64% intestinal), confirming its high instability in acidic and enzymatic environments. The enhanced stability of glycosylated forms suggests that conjugation with sugars exerts a protective effect, delaying oxidation and degradation processes in the gastrointestinal tract. These findings are consistent with those reported also by Cattivelli et al. [32], who demonstrated that after in vitro digestion of red onions, only 38.6% of phenolic compounds remained bioaccessible, with significant degradation of quercetin-hexosides due to hydrolysis and oxidation. This aligns closely with the 49.09% intestinal recovery observed in our red onion samples. Similarly, Mihaylova et al. [33] reported substantial decreases in total polyphenols and the anthocyanins in red fruit juices after intestinal digestion, likely due to the mildly alkaline pH and enzymatic hydrolysis, with bioaccessibility rates of 13–26% for total polyphenols and below 1.1% for anthocyanins.

In the present study, anthocyanins identified in red onion peels, including cyanidin 3-glucoside, cyanidin 3-(6″-malonylglucoside), and cyanidin glycosides, showed moderate gastric stability (57–80%) but were significantly degraded during the intestinal phase (~30%). These results confirm that anthocyanins are highly sensitive to pH and digestive enzymes, as they degrade rapidly under alkaline conditions (pH > 5.5). According to Koh et al. [34], anthocyanins such as delphinidin and cyanidin degrade quickly under intestinal conditions, unless stabilized by macromolecules such as pectins, which can form protective complexes through hydrogen bonding and electrostatic interactions. Although onion peels naturally lack such pectin matrices, future formulation strategies could consider co-encapsulation with pectin or dietary fibers to enhance anthocyanin stability and promote their delivery to the colon. Interestingly, protocatechuic acid and taxifolin glycoside showed recoveries exceeding 100% in both onion varieties, particularly in red onion onions, suggesting that they may form through the oxidative or enzymatic degradation of more complex polyphenols, such as quercetin monoglucosides, during digestion. This transformation phenomenon has been previously described by Cattivelli et al. [35], who reported the formation of simpler phenolic acids after thermal and enzymatic treatment of red onions. From a physiological perspective, these degradation products are not necessarily negative, as low-molecular-weight phenolic acids such as protocatechuic acid can still exert antioxidants, anti-inflammatory, and antimicrobial effects, and may represent the main bioaccessible fraction reaching the colon [35].

When comparing the two onion varieties, although red onion peels contained a higher initial total polyphenols content (28.44 mg/g vs. 15.61 mg/g), yellow onion peels demonstrated greater stability and recovery after intestinal digestion (72.73% vs. 49.09%). This could be attributed to a higher prevalence of glycosylated flavonols (e.g., quercetin mono- and diglucosides) in yellow onions, which are generally more resistant to digestive degradation. Mihaylova et al. [33] also emphasized that glycosylated phenolics tend to be more stable than aglycones under digestive conditions, thus contributing to enhanced bio accessibility.

### 3.3. Intestinal Accumulation and Bioavailability of Red and Yellow Onion Peels’ Polyphenols

Bioactive compounds naturally found in plants, particularly polyphenols, exhibit good bioaccessibility (the percentage of a compound released from the ingested matrix during gastrointestinal digestion); however, their bioavailability (presence in the bloodstream) remains limited. In the human digestive tract, dietary polyphenols are not easily absorbed in the small intestine, but they can reach the colon, where they undergo biochemical transformations (hydrolysis, cleavage, reduction, and deglycosylation) and metabolism by gut microbiota, resulting in the production of low-molecular-weight derivatives. These final products can be easily absorbed, thereby improving both bioavailability and health-promoting effect [36].

To assess the absorption and the bioavailability of polyphenols present in red and yellow onion peels, the Caco-2 cell model was employed. Differentiated Caco-2 cells, resembling enterocytes, possess extra- and intracellular esterases that are able to de-esterify hydroxycinnamate and diferulate esters that may contribute to metabolism [37,38]. Additionally, they exhibit low expression of certain transport-related enzymes (such as lactase-phlorizin hydrolase and cytosolic β-glucosidase) compared to ex vivo intestinal samples. Nevertheless, they share key physiological properties with the human intestinal epithelium, particularly that of the colon. As a result, this model is widely used to study both the absorption and metabolism of polyphenolic compounds [16,39,40].

The polyphenolic compounds present in the extracts for absorption and bioavailability studies belong to the following classes: flavonols (quercetin, both in aglycone form and as mono- and diglycosylated derivatives); protocatechuic acid; flavanonols (taxifolin); and anthocyanins (cyanidin derivatives in glycosylated form), which are exclusively present in red onion peels.

**Figure 3:**
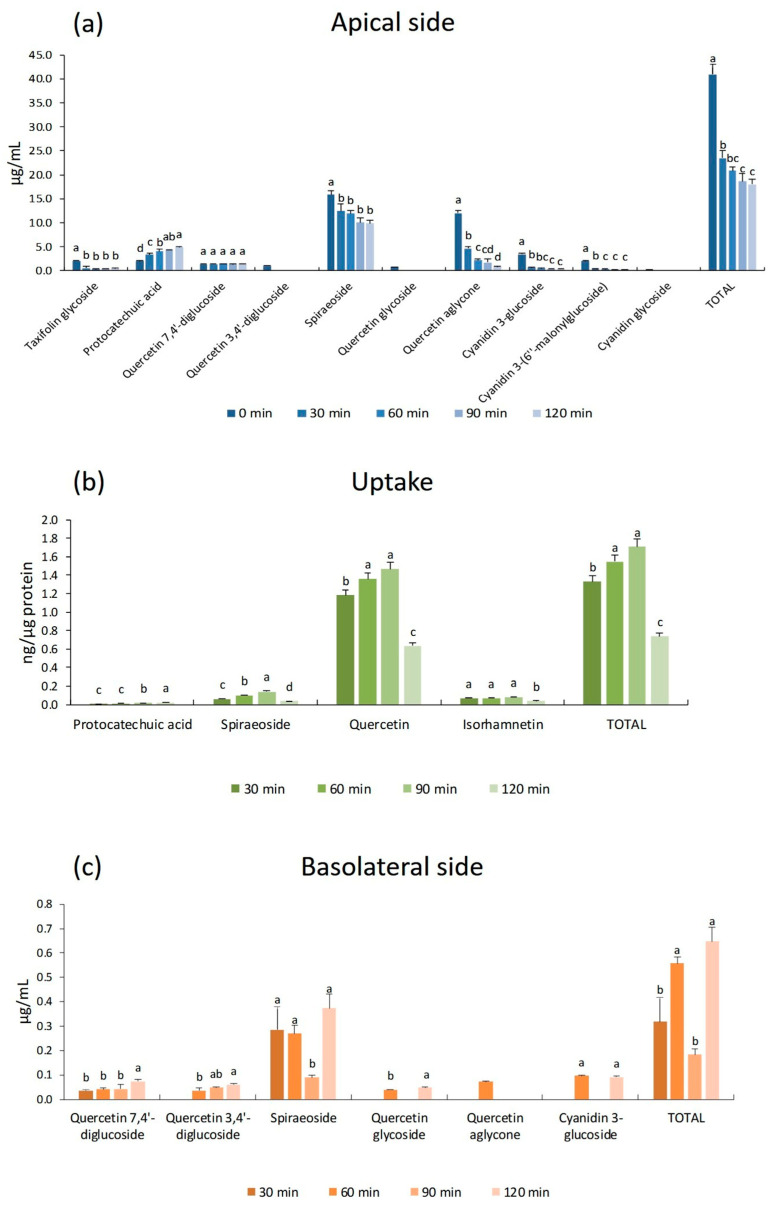
Concentration of the main polyphenols of red onion peels identified by HPLC-DAD analysis in (**a**) apical compartment, (**b**) Caco-2 cells, and (**c**) basolateral compartment after 30, 60, 90, and 120 min of incubation. Different letters above the bars indicate significant differences among incubation times for the same compound, as determined by repeated-measures ANOVA, followed by Tukey’s post hoc test (*p* < 0.05).

In Figure 3, the concentrations of the main identified polyphenols of red onion peels in cells and in culture media (apical and basolateral) are reported. The amounts of phenolic compounds detected on the apical side provided insights into their stability within the culture medium, whereas the concentration of polyphenols recovered on the basolateral side reflected their transport dynamics and metabolic transformation over time. As illustrated in Figure 3a, after 30 and 60 min of incubation, approximately 57% and 51% of the initial polyphenol contents were recovered, respectively. A gradual decrease was subsequently observed at 90 and 120 min, reaching a final recovery of 44% with respect to the initial concentration. Notably, a pronounced reduction in quercetin aglycone was recorded during the incubation period, with its relative abundance decreasing from 38% at 30 min to 7% at 120 min. This decline was accompanied by a parallel increase in the levels of protocatechuic acid over time, suggesting an ongoing metabolic conversion process of quercetin [35]. Also, the spiraeoside concentration was reduced over time, from 78% (30 min) to 62% (120 min), together with all the polyphenols identified in red onion peels, apart from quercetin 7,4′-diglucoside, which resulted in being quite stable.

The intracellular accumulation of polyphenols in Caco-2 monolayers exposed to red onion peels extract was monitored over 120 min, and the reported results highlighted that the incubation time influenced the polyphenol uptake (Figure 3b). The maximum of cellular accumulation of polyphenols for red onion extract (1715 ng/µg protein) was reached after 90 min. Among the identified compounds absorbed from the Caco-2 monolayer, quercetin was the most abundant, reaching a maximum of 1.468 ng/µg protein, followed by spiraeoside, isorhamnetin, and protocatechuic acid. After 120 min, an overall reduction of the identified compounds was recorded, with a total cellular accumulation of 0.743 ng per μg protein. Protocatechuic acid was detected at relatively low concentrations (0.013 ng/µg protein at 30 min), and it exhibited a modest, non-significant increase over time. Spiraeoside levels, conversely, showed a progressive increase throughout the incubation period, reaching a maximum concentration of 0.142 ng/µg protein at 90 min. Notably, isorhamnetin was detected exclusively in red onion treatments, with concentrations ranging from 0.072 to 0.080 ng/µg protein up to 90 min, followed by a decrease to 0.040 ng/µg protein at 120 min.

The results obtained for red onion peels revealed that quercetin aglycone represented the most efficiently absorbed compound, consistent with previous findings on quercetin uptake from onions [39]. It has been demonstrated that Caco-2 cells hydrolyze quercetin glycosides through interactions with SGLT1 and cytosolic β-glucosidase, which may explain the elevated quercetin levels observed in this study [41]. Moreover, the detection of isorhamnetin, a methylated metabolite of quercetin generated via catechol-O-methyltransferase activity in Caco-2 cells, underscores the metabolic competence of these cells toward flavonoid biotransformation [42]. Importantly, isorhamnetin has been reported to enhance glucose uptake and may play a key role in glucose homeostasis and hyperglycemia prevention through JAK2/STAT-mediated signaling pathways [43]. The bioavailability results of red onion peels’ polyphenols (Figure 3c) showed a time-dependent trend, with an increase in the bioavailable fraction related to the exposure time (30, 60, 90, and 120 min). This effect was mainly evident for glycosylated quercetins, which showed the highest bioavailability value, ranging from 2.3 to 6.4%, at 120 min; meanwhile, quercetin aglycone was poorly bioavailable (0.6% at 60 min). As reported in numerous studies, quercetin aglycone is lipophilic and mainly absorbed by intestinal epithelial cells through passive diffusion. However, the efficiency of this transport route is lower than that of the SGLT1 and GLUT2 transporter systems utilized by glycosylated molecules, which benefit from their higher water solubility [10,44]. Taxifolin glycoside, one of the characteristic compounds of onions, was not detected in the basolateral, probably for its chemical property. In fact, the taxifolin has slight solubility (0.1% at room temperature), and as reported in other studies, it is difficult for taxifolin to be absorbed and metabolized by the body, thus significantly limiting its bioavailability and efficacy [45]. Anthocyanin compounds also show low bioavailability, although cyanidin 3-glucoside was present in the basolateral side at 60 and 120 min. The low quantities of anthocyanins found in the basolateral side may be due to the low stability of these molecules at neutral pH and to the long incubation time [46]. Furthermore, the presence of sugars and the degree of glycosylation also seem to positively influence anthocyanins’ stability and bioavailability.

**Figure 4:**
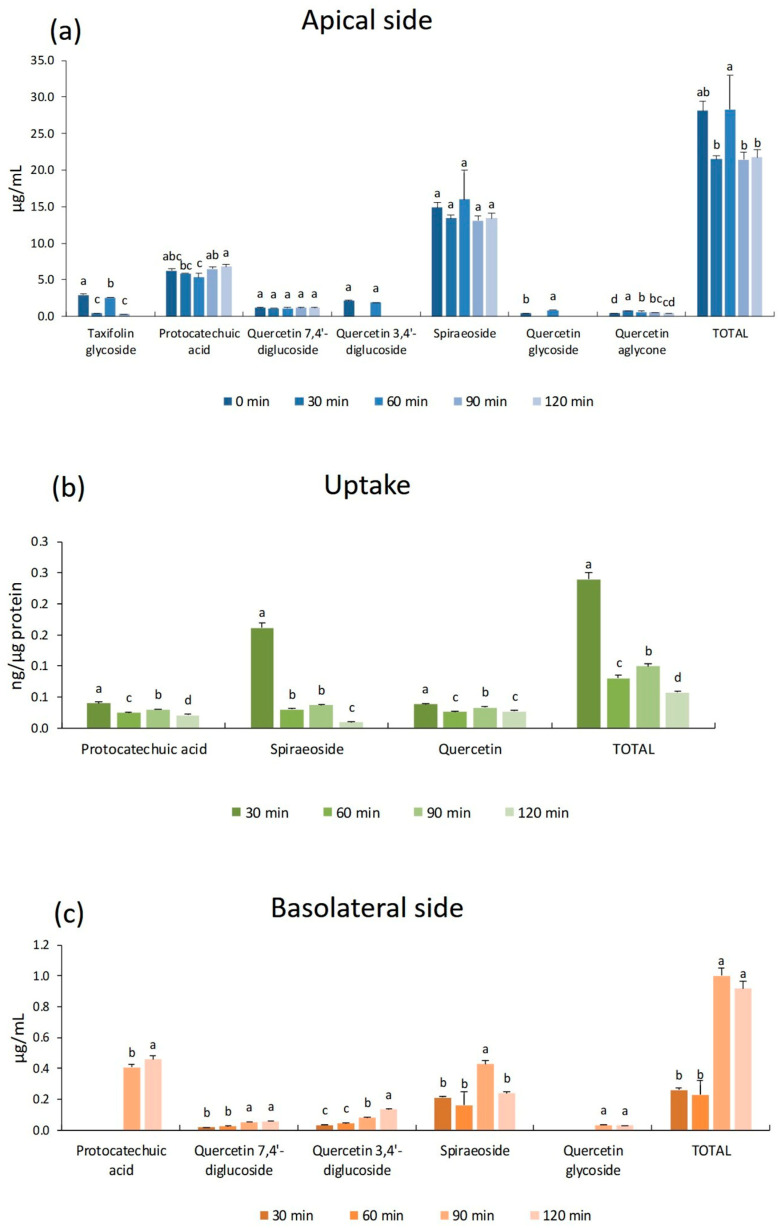
Concentration of the main polyphenols of yellow onion peels identified by HPLC-DAD analysis in (**a**) apical compartment, (**b**) Caco-2 cells, and (**c**) basolateral compartment after 30, 60, 90, and 120 min of incubation. Different letters above the bars indicate significant differences among incubation times for the same compound, as determined by repeated-measures ANOVA, followed by Tukey’s post hoc test (*p* < 0.05).

Figure 4 shows the concentrations of the main identified polyphenols from yellow onion peels in cells and in the culture media (apical and basolateral). Figure 4a illustrates the polyphenols and their stability over the selected incubation times. The total concentration of polyphenols remains relatively stable throughout the incubation period, ranging between 21.5 μg/mL and 28.3 μg/mL. A slight increase is observed at 60 min (28.3 μg/mL), followed by a return to levels close to the initial measurement (≈21.7 μg/mL at 120 min). Regarding individual compound, protocatechuic acid and quercetin diglucosides (7,4′- and 3,4′-) showed consistent stability, while spiraeoside exhibited a slight transient increase at 60 min. In contrast, taxifolin glycoside and quercetin glycoside displayed irregular trends, suggesting possible transformation or instability. Quercetin aglycone showed an initial rise, followed by a gradual decline, likely due to transient release from glycosides and subsequent degradation or oxidation. This behavior may reflect temporary release or conversion between conjugated and free forms of quercetin derivatives. The detected presence observed indicates that these compounds can persist under the tested conditions, which is important for assessing their potential bioavailability and antioxidant activity in cell-based studies.

The yellow onion peels’ polyphenols’ cellular accumulation is shown in Figure 4b. Protocatechuic acid was detected at higher concentrations in respect to red onion samples, with a maximum value of 0.050 ng/µg protein at 30 min. Its levels subsequently declined gradually throughout the incubation period. In contrast, spiraeoside displayed an opposite temporal pattern to that observed in the red onion treatment: its concentration was highest at 30 min (0.162 ng/µg protein) and then decreased sharply over time. Caco-2 absorption data indicated that spiraeoside (Figure 4b) was the most efficiently absorbed polyphenol from yellow onion peels. Spiraeoside, a natural quercetin-4′-O-glucoside, possesses well-documented antioxidant and anti-inflammatory activities and has also been identified as a potent anticancer compound against HeLa cells [47].

The bioavailability data of polyphenols present in yellow onion peels (Figure 4c) showed the same trends as red onion, especially for the flavonol class. In fact, the bioavailability of the glycosylated forms was generally time-dependent, and quercetin 4-O glucoside (spiraeoside) was the most bioavailable at both 90 and 120 min, reaching about 8%. The quercetin aglycone, instead, was not detected on the basolateral side, thus confirming its low bioavailability. In addition, the protocatechuic acid was one of the most abundant compounds; its bioavailability was high after long incubation time (90 and 120 min, 6.8% and 7.7%, respectively). These results agree with the results of Paesa et al. [28], who demonstrated that the protocatechuic acid has good intestinal permeability, reaching high concentrations on the basolateral side. Different results have been obtained for the red onion extract, probably because the tested quantities were approximately three times lower than those present in the yellow onion extract.

In order to provide additional insight for the prediction of absorption of polyphenols typical of onion peels, the values obtained on its transport and accumulation were used to calculate the Papp, which represents the apical-to-basolateral transport rate, across the epithelial barrier, normally used for drugs [48] and applied to others phenolic compounds [49] and vegetable matrices, such as artichoke and table olives [16,50]. The Papp values for red onion peels ranged from 1.1 × 10^−6^ cm·s^−1^ for quercetin to 9.2 × 10^−6^ cm·s^−1^ for quercetin glycosides, indicating a potential bioavailability of all the identified compounds. The literature evidence suggests that in vivo absorption can be estimated based on the Papp value: Papp ≤ 1 × 10^−6^ cm·s^−1^ corresponds to a low absorption rate (0–20%), 1 × 10^−6^ < Papp ≤ 10 × 10^−6^ cm·s^−1^ indicates moderate absorption (20–70%), and Papp > 10 × 10^−6^ cm·s^−1^ reflects high absorption (70–100%) [51,52].

The results presented in these studies demonstrated that the glycosylated compounds are moderately absorbed mainly by glucose transporters; the low quercetin Papp value is in line with its poor bioavailability, as already demonstrated. Interestingly, the data highlight the potential for bioavailability of cyanidin 3-glucoside (Papp 5.1 × 10^−6^ (cm·s^−1^), indicating a moderate level of absorption. Similar considerations are also reported for yellow onion peels, with all the compounds moderately bioavailable: 5.5 spiraeoside < 5.9 quercetin 7,4′-diglucoside < 6.1 quercetin 3,4′-diglucoside < 8.4 protocatechuic acid < 9.9 × 10^−6^ cm·s^−1^ quercetin glycoside.

### 3.4. Antioxidant Activity of Red and Yellow Onion Polyphenols

#### 3.4.1. DPPH Radical-Scavenging Activity and FRAP

The consumption of fruits and vegetables is inversely associated with age-related diseases, primarily due to the presence of antioxidant compounds, especially those with phenolic structures, which are effective radical scavengers. Among the various chemical methods developed to assess antioxidant potential, DPPH and FRAP assays are the most widely used.

The DPPH assay measures the ability of antioxidants to reduce the DPPH* radicals, either by monitoring the decrease in absorbance or through electron paramagnetic resonance (EPR) detection. The radical-scavenging activity is typically expressed as the percentage of DPPH* inhibition or as the IC_50_ value (the concentration of antioxidant needed to cause a 50% inhibition of radical activity). Lower IC_50_ values indicate higher antioxidant activity [53]. In regard to the hydroalcoholic extracts of onion peels, both onion varieties demonstrated a good ability to scavenge DPPH* radicals, with red onion showing significantly higher antioxidant activity (IC_50_ = 54.6 ± 0.2 µg/mL) compared to yellow onion (EC_50_ = 59.1 ± 0.8 µg/mL) (*p* < 0.05). These results align with the existing literature reporting stronger DPPH scavenging activity in red onions, mainly attributed to their higher concentrations of phenolic compounds, such as flavonoids, quercetin, and anthocyanins. Studies on Moroccan ecotypes reported similarly lower DPPH IC_50_ values in red onions compared to yellow onions, correlated to their higher polyphenols content [54,55].

The FRAP assay quantifies the antioxidant potential of a sample by measuring its capacity to reduce ferric (Fe^3+^) to ferrous (Fe^2+^) ions, resulting in the formation of a colored Fe^2+^–TPTZ complex that can be detected spectrophotometrically. Thus, while DPPH specifically evaluates radical-scavenging efficiency, FRAP primarily reflects the overall reducing power of a sample. In relation to FRAP, red onion peels exhibited a significantly higher FRAP value (426.5 ± 43.2 µmol TE/g DW) compared to yellow ones (229.7 ± 22.3 µmol TE/g DW) (*p* < 0.05), indicating a markedly greater reducing capacity. This difference in activity can be largely attributed to the variation in phytochemical profiles between the two onion types, as demonstrated by other authors [56].

Overall, the combined evidence from the DPPH and FRAP assays confirms that red onion peels exhibit a stronger antioxidant potential than yellow onion peels. This enhanced activity of red onion is primarily attributed to higher levels of anthocyanins, particularly cyanidin derivatives, along with quercetin and its glycosides, which have been extensively studied for their strong antioxidant properties. In contrast, yellow onions primarily contain quercetin derivatives but lack the additional anthocyanin content that contributes to the deeper pigmentation of red onions and potentially to their enhanced radical-scavenging performance. These findings are in agreement with the observations of Slimestad et al. (2007) and Lanzotti (2006) [57,58], who reported that red onion varieties generally possess richer phenolic profiles and stronger antioxidant properties than yellow cultivars. However, it is worth noting that the absolute values obtained for DPPH and FRAP assays can vary considerably among studies, as they are strongly influenced by several methodological and biological factors, including the extraction protocol (solvent type, time, and temperature), the onion cultivar, and the environmental conditions during growth. Despite this variability, the relative trend observed, red onion exhibiting higher antioxidant capacity than yellow onion, remains consistent with previous findings in the literature.

#### 3.4.2. Toxicity Assessment

The MTT test was applied to evaluate the potential toxic effect of hydroalcoholic extracts of onions peels on intestinal cell model and to select the higher non-toxic concentration of total polyphenols for in vitro antioxidant activity.

The viability results were expressed as the percentage of cell viability relative to the solvent control (% viability) for each tested concentration of total polyphenols after 3 h of exposure. When cellular metabolic activity was assessed through succinate dehydrogenase activity, a reduction in enzyme activity and, consequently, in cell viability, was observed at the highest concentrations of red onion extract (120 µg/mL, corresponding to a 44% inhibition of cell proliferation) and yellow onion extract (60 µg/mL, corresponding to a 34% inhibition). In contrast, the exposure to the other tested polyphenols concentrations did not show significant differences in viability compared to solvent control. These findings suggest a dose-dependent inhibitory effect on cellular metabolism, as measured by the MTT assay. The observed decrease in cell viability at higher concentrations of red and yellow onion peels extracts is consistent with the previous literature indicating that elevated levels of polyphenols can exert cytotoxic effects. In fact, several studies have demonstrated that polyphenols are widely recognized for their antioxidant and anti-inflammatory properties at low-to-moderate concentrations, but at higher doses, they may exert pro-oxidant activity. This can lead to the generation of reactive oxygen species mitochondrial dysfunction, and ultimately cellular damage or apoptosis [59,60].

#### 3.4.3. Cellular Antioxidant Activity Assay

To evaluate the antioxidant potential of onion peels polyphenols, the Cellular Antioxidant Activity (CAA) assay was employed. This cell-based assay considers the uptake, distribution, and protective efficiency of antioxidants under physiological conditions, providing a more biologically relevant measure than purely chemical assays. Red and yellow onion polyphenols were tested for their ability to reduce intracellular-induced ROS in intestinal cells. The results (Table 3) demonstrate a clear dose-dependent antioxidant response for both red and yellow onion extracts.

For red onion, CAA values increased from 42 ± 1.3 units at 1 µg/mL to 84 ± 1.0 units at 30 µg/mL, while yellow onion extracts showed an increase from 44 ± 3.9 units at 0.8 µg/mL to 75 ± 1.0 units at 15 µg/mL. These findings confirm that polyphenol-rich onion peel extracts effectively quench intracellular ROS in intestinal cells. The median effective dose (MED), representing the lowest concentration eliciting a significant antioxidant response, was lower for yellow onion (1.03 ± 0.23 µg/mL) than for red onion (1.69 ± 0.42 µg/mL). Although there is no statistical difference between the MED values of red and yellow onion, the results indicated a slightly higher cellular efficacy for yellow onion polyphenols. Previous studies support these observations. Mearefati et al. [8] reported that onion polyphenols exert antioxidant effects via stabilization of cellular membranes, ROS scavenging, and reduction of lipid peroxidation. Similarly, Paesa et al. [28] demonstrated that onion extracts prevented H_2_O_2_-induced ROS in Caco-2 cells without significantly altering basal redox balance.

The observed differences between chemical antioxidant assays (DPPH and FRAP) and the CAA results can be attributed to their distinct principles. Chemical assays measure direct radical-scavenging ability, favoring lipophilic, structurally simple compounds such as quercetin aglycone, abundant in red onion peels [61]. In contrast, the CAA assay evaluates antioxidant activity in a physiological context, accounting for cellular uptake, metabolism, efflux, and intracellular ROS scavenging [25]. Red onion’s higher quercetin aglycone content explains its superior performance in chemical assays, whereas yellow onion, richer in quercetin glycosides, exhibits greater solubility and cellular absorption [44,57]. In fact, as shown in the uptake results’ section, in Caco-2 cells treated with yellow onion peel extract, the highest intracellular accumulation of polyphenols was observed after 30 min (0.250 ng/µg protein), and spiraeoside, a quercetin glycoside, was the predominant compound detected (0.162 ng/µg protein). Furthermore, metabolizing enzymes and efflux transporters in Caco-2 cells may selectively modulate intracellular retention of glycosylated polyphenols) [62]. Consequently, while red onion demonstrates higher chemical antioxidant capacity, yellow onion shows superior cellular efficacy, highlighting the importance of cell-based assays for evaluating dietary antioxidants.

#### 3.4.4. GSH Determination

The fluorimetric measurement of intracellular GSH was performed to assess the effect of onion polyphenols on cellular redox status under basal conditions, without any external induction of oxidative stress. Results are expressed as a percentage of fluorescence relative to the control.

For red onion, the highest tested concentration of polyphenols (28.40 µg/mL) induced a significant (*p* < 0.05) increase in intracellular GSH content in the intestinal cell line. The fluorescence intensity of MCB in the presence of red onion polyphenols was approximately twice that of the control. In contrast, lower polyphenol concentrations did not significantly affect GSH fluorescence levels, which remained comparable to those of the control samples. Similar results were observed for yellow onion: only 15.05 µg of total polyphenols/mL induced a fourfold enhancement of MCB fluorescence, reflecting a marked increase in intracellular GSH levels. Our results demonstrate that polyphenols enhance the cellular defense response against oxidative stress. In particular, the administration of red and yellow onion polyphenols led to a significant increase in intracellular GSH levels, as reflected by MCB fluorescence, indicating a strengthened antioxidant capacity.

Glutathione, the most abundant low-molecular-weight thiol in cells, plays a central role in maintaining redox homeostasis and protecting against oxidative damage and xenobiotic toxicity. Reduced intracellular GSH has been associated with oxidative stress and the progression of various pathological conditions, including neurodegeneration, inflammation, and infections [63]. Our findings align with this evidence, suggesting that polyphenol-induced elevation of GSH may contribute to cellular protection. Furthermore, the ROS-mediated intrinsic apoptotic pathway underscores the importance of intracellular GSH in regulating cell fate. Cells with higher GSH content exhibit increased resistance to apoptosis, highlighting the potential of polyphenols to modulate apoptotic susceptibility under oxidative stress conditions [64].

## 4. Conclusions

Onion peels are a valuable source of bioactive polyphenols, particularly quercetin derivatives and, in red cultivars, anthocyanins. The red onion “*Rossa di Tropea*” peels were found to contain a significantly higher total polyphenol content (28.44 mg/gr DW) compared to the yellow “*Recas*” peels (15.61 mg/gr DW). Although gastrointestinal digestion reduced their overall content, digestive stability showed differences between cultivars, with a higher intestinal recovery for yellow peels (72.7%) than for red peels (49.1%). Glycosylated flavonols proved to be more stable and exhibited moderate intestinal transport (Papp = 1.1–9.9 × 10^−6^ cm·s^−1^), while quercetin aglycone and anthocyanins had limited recovery. Studies on Caco-2 cells confirmed a modest but measurable bioavailability for several compounds, with quercetin glycosides and protocatechuic acid showing the highest permeability.

The antioxidant evaluation highlighted two complementary behaviors: red onion peels demonstrated a superior chemical radical-scavenging capacity, while yellow onion peels seemed to be slightly more effective in cellular assays, as demonstrated by CAA and GSH assays. These findings suggest that onion peel extracts can reinforce intestinal redox homeostasis despite their limited absorption.

From an application perspective, onion peel polyphenols represent promising candidates for nutraceutical and functional food development within circular economy strategies. However, targeted formulation approaches—such as encapsulation, complexation with dietary fibers, or controlled-release systems—are needed to improve their gastrointestinal stability and bioavailability. Future research should include colonic fermentation models and in vivo studies to elucidate the metabolic fate and biological relevance of onion-derived phenolic metabolites.

## Figures and Tables

**Table 1:** Polyphenol characterization and gastrointestinal stability (%) of red onion peels “*Rossa di Tropea*”.

	Polyphenols	Digestive Stability (%)	
	(mg/g DW)	Gastric %	Intestinal %
*Phenolic acid*			
Protocatechuic acid	0.52 ± 0.03	>100.00	>100.00
*Flavonoid glycosides* and *aglycone*			
Taxifolin glycoside	0.34 ± 0.02	>100.00	>100.00
Quercetin 7,4′-diglucoside	0.69 ± 0.03	84.61 ± 0.82 ^a^	82.20 ± 10.42 ^a^
Quercetin 3,4′-diglucoside	0.48 ± 0.02	97.96 ± 0.29 ^a^	84.37 ± 7.76 ^b^
Spiraeoside	8.08 ± 0.40	99.51 ± 3.50 ^a^	39.97 ± 6.96 ^b^
Quercetin glycoside	0.42 ± 0.02	78.82 ± 3.61 ^a^	45.63 ± 8.60 ^b^
Quercetin	14.07 ± 0.70	32.34 ± 3.97 ^a^	15.37 ± 2.55 ^b^
*Anthocyanins*			
Cyanidin 3-glucoside	1.96 ± 0.10	80.08 ± 4.58 ^a^	30.39 ± 3.91 ^b^
Cyanidin 3-(6″-malonylglucoside)	1.65 ± 0.08	62.25 ± 4.45 ^a^	32.61 ± 1.99 ^b^
Cyanidin glycoside	0.22 ± 0.01	57.94 ± 2.28 ^a^	30.98 ± 1.86 ^b^
Total identified polyphenols	28.44 ± 1.42	63.39 ± 3.69 ^a^	49.09 ± 4.29 ^b^

Data are expressed as mean ± standard deviation of three independent experiments. ^a,b^ Values in the same row with different letters differ significantly (*p* < 0.05).

**Table 2:** Polyphenol characterization and gastrointestinal stability (%) of yellow onion peels “*Recas*”.

	Polyphenols	Digestive Stability (%)	
	(mg/g DW)	Gastric %	Intestinal %
*Phenolic acid*			
Protocatechuic acid	2.13 ± 0.11	90.32 ± 4.41 ^b^	>100.00 ^a^
*Flavonoid glycosides* and *aglycone*			
Taxifolin glycoside	0.49 ± 0.02	>100.00	>100.00
Quercetin 7,4′-diglucoside	0.56 ± 0.03	90.74 ± 0.97 ^a^	80.06 ± 2.63 ^b^
Quercetin 3,4′-diglucoside	1.02 ± 0.05	97.40 ± 0.25 ^a^	84.42 ± 2.94 ^b^
Spiraeoside	8.35 ± 0.42	81.11 ± 6.54 ^a^	36.76 ± 3.49 ^b^
Quercetin glycoside	0.22 ± 0.01	69.36 ± 4.67 ^a^	52.52 ± 3.82 ^b^
Quercetin	2.85 ± 0.14	3.44 ± 0.56 ^b^	15.64 ± 0.96 ^a^
Total identified polyphenols	15.61 ± 0.78	70.92 ± 4.21 ^a^	72.73 ± 2.49 ^a^

Data are expressed as mean ± standard deviation of three independent experiments. ^a,b^ Values in the same row with different letters differ significantly (*p* < 0.05).

**Table 3:** CAA values obtained from hydroalcoholic extracts of onion peels on Caco-2 cells.

Red Onion Peels		Yellow Onion Peels	
Total Polyphenols (µg/mL)	CAA Units	Total Polyphenols (µg/mL)	CAA Units
30	84 ± 1.1	15	75 ± 1.0
12	76 ± 2.4	6	68 ± 3.0
6	68 ± 3.2	3	64 ± 3.5
3	58 ± 4.2	2	58 ± 2.7
2	50 ± 4.2	1.5	54 ± 2.5
1.5	48 ± 3.7	1.2	53 ± 0.2
1.2	43 ± 3.4	1.0	50 ± 0.8
1	42 ± 1.3	0.8	44 ± 3.9
0.9	40 ± 4.5	0.6	42 ± 3.4
MED 1.69 ± 0.42 µg/mL		MED 1.03 ± 0.23 µg/mL	

Data are expressed as mean ± standard deviation from three independent experiments. MED: median effective dose mathematically calculated from CAA values.

## Data Availability

The original contributions presented in this study are included in the article. Further inquiries can be directed to the corresponding authors.

## Abbreviations

DPPH	2, 2-diphenyl-1-picrylhydrazyl
FRAP	Ferric ion-reducing antioxidant power
TEER	Trans Epithelial Electrical Resistance
Papp	Permeability coefficient
TE	Trolox equivalent
DW	Dry weight
CAA	Cellular Antioxidant Activity
GSH	Reduced glutathione
ROS	Reactive oxygen species
